# Ultrasound and Microwave Assisted Extraction of *Opuntia* Fruit Peels Biocompounds: Optimization and Comparison Using RSM-CCD

**DOI:** 10.3390/molecules24193618

**Published:** 2019-10-08

**Authors:** Bruno Melgar, Maria Inês Dias, Lillian Barros, Isabel C.F.R. Ferreira, Antonio D. Rodriguez-Lopez, Esperanza M. Garcia-Castello

**Affiliations:** 1 Institute of Food Engineering for Development (IIAD), Universitat Politècnica de València, Camino de Vera, s/n CP, 46022 Valencia, Spain; bruno.melgarc@gmail.com; 2 Centro de Investigação de Montanha (CIMO), Instituto Politécnico de Bragança, Campus de Santa Apolónia, 5300-253 Bragança, Portugal; maria.ines@ipb.pt (M.I.D.); lillian@ipb.pt (L.B.); 3 Institute for Industrial, Radiophysical and Environmental Safety (ISIRYM), Universitat Politècnica de València, Camino de Vera, s/n CP, 46022 Valencia, Spain; anrodlo@iqn.upv.es

**Keywords:** *Opuntia*, by-products, phenolic compounds, betalains, extraction optimization, response surface methodology (RSM)

## Abstract

Ultrasound-assisted extraction (UAE) and microwave-assisted extraction (MAE) of bioactive compounds, peels from *Opuntia engelmannii* cultivar (cv.) Valencia were optimized by response surface methodology. Randomized extraction runs were performed for each of the technologies employed in order to build effective models with maximum (bioactive molecules content and yield) and minimum (antioxidant activity) responses. A 5-level, 4-factor central composite design was used to obtain target responses as a function of extraction time (*t*), solid to liquid ratio (S/L), methanol concentration (*metOH*), and temperature (*T*). Specific response optimization for each technology was analyzed, discussed, and general optimization from all the responses together was also gather. The optimum values for each factor were: *t* = 2.5 and 1.4 min, S/L = 5 and 5 g/L, *metOH* = 34.6 and 0% of methanol and *T* = 30 and 36.6 °C, achieving maximum responses of 201.6 and 132.9 mg of betalains/g, 13.9 and 8.0 mg of phenolic acids/g, 2.4 and 1.5 mg of flavonoids/g, 71.8% and 79.1% of extractable solid and IC_50_ values for the antioxidant activity of 2.9 and 3.6, for UAE and MAE, respectively. The present study suggested UAE as the best extraction system, in order to maximize recovery of bioactive compounds with a high antioxidant activity.

## 1. Introduction

The actual food market trends for a diversification of their ingredients, and consumers have a growing consciousness for healthier food products; these two aspects have made an important transformation in the food industries, which is looking beyond the flavor/nutrition balance in their products, extending their awareness in the usage of organic products and natural ingredients as part of their new formulations [1,2]. Therefore, exponential growth in research and development of new alternatives to synthetic additives (such as colorants or bioactive compounds) have been studied in recent years [3]. Incorporation of additives can be listed by their important impact on the final products, such as (1) maintaining or improving safety and freshness, (2) improving or maintaining nutritional value and/or (3) improving taste, texture, and appearance [4]. Furthermore, transversal interest in food security and environmental protection has also encouraged the development in by-products recovery and utilization. 

*Opuntia* spp., grows in arid and semiarid environments and belongs to the Cactaceae family. Prickly pear is a meaningful source of natural pigments, such as betalains and bioactive compounds, such as polyphenols, which have been recognized to have health-promoting effects and are considered an interesting source of pharmacologically active phytochemicals, whose involvement in antimicrobial and antioxidant processes have been demonstrated previously [5]. Despite the potential of these natural biomolecules and their possible application in the industrial sectors, the development of more efficient processes for their recovery remains challenging and is a current hot topic.

Several studies have been carried out to improve the extraction of these compounds from different plant materials throughout conventional, ultrasound, or microwave assisted extraction [6,7,8,9], but a more efficient and robust analysis in *Opuntia* spp. peels are needed to achieve superior quality phytochemicals at lower processing costs and in an environmentally friendly manner.

This work will also address some of the six principles of green energy [10], comprehending the following points: (1) Utilization of fruit byproducts as natural renewable sources, which could also be aligned perfectly with other waste processes, such as plant milking; (2) percentage and volume reduction of solvents employed; (3) reduction of energy consumption by process intensification using UAE and MAE technologies as alternatives to conventional extraction; and (4) time and operational units reduction due to the innovative technologies applied.

Ultrasound and microwave assisted extractions (UAE and MAE, respectively) are emerging technologies increasingly used in extraction industries. UAE is a process that uses acoustic energy (a mechanical energy i.e., it is not absorbed by molecules, but is being transmitted throughout the medium) and solvents to extract target compounds from various plant matrices [11]. Ultrasound is transmitted through a medium via pressure waves by inducing vibrational motion of the molecules which alternately compress and stretch the molecular structure of the medium due to a time-varying pressure. While, microwaves, heat up the molecules of any object by a dual mechanism of ionic conduction and dipole rotation, both techniques end up by disrupting the cell walls and releasing the compounds of interest to the extracting solvent [12].

The conventional extraction methodology of changing one variable at a time to study the effects of variables on the responses analyzed is an exhausting and expensive task, especially for multivariable systems. Thus, in order to obtain a significant model of different variables performing a minimum number of experiments, statistical design experiments become necessary. Central composite design (CCD) along with response surface methodology (RSM) are efficient and flexible tools, which provide sufficient data on the modelling of multivariable systems, minimizing experimental errors and reducing significantly the number of experiments needed [13].

To our knowledge, there is scarce information about the recovery of betalains and phenolic compounds from *Opuntia* spp. peels using UAE and MAE techniques. Therefore, the main objective of this study was to optimize these two extraction systems using a RSM methodology, in order to identify which of the employed techniques could be potentially established to maximize molecules recovery from *Opuntia* by-products.

## 2. Results and Discussion

### 2.1. Phytochemical Identification

**Figure 1:**
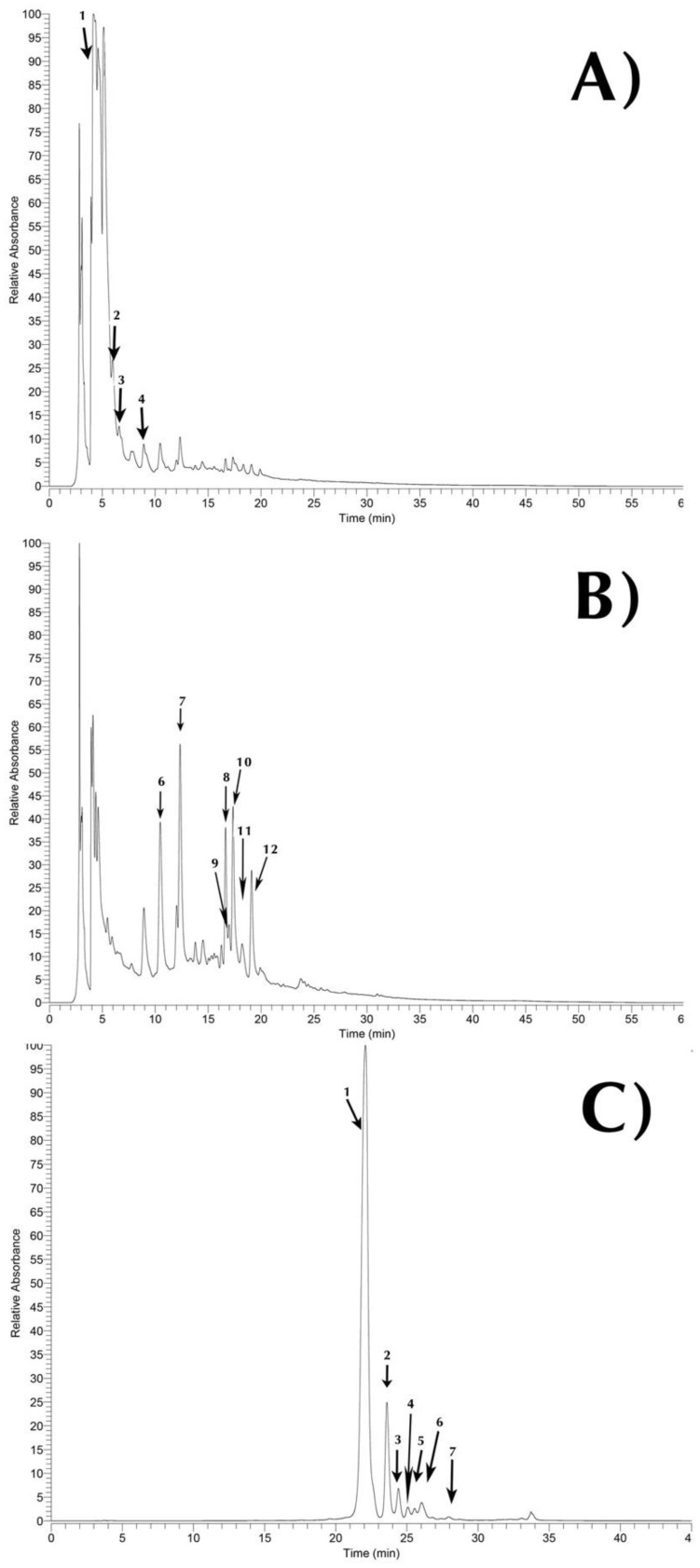
HPLC chromatogram of *Opuntia engelmannii* cv. Valencia peels phenolic and betalainic profile recorded at 280 nm Ph1–Ph4 phenolic acids (**A**), 370 nm Fv1-Fv8 Flavonoids (**B**), and 535 nm Bc1-Bc7 Betalains (**C**). Peak numbers correspond to the compounds described in Table 1.

Table 1. presents the peak characteristics (abbreviation used, retention time, wavelength of maximum absorption, molecular ion, and main fragment ions observed in MSn) and tentative identification of the phytochemicals (phenolic and betalain compounds) present in the hydromethanolic extracts of *Opuntia engelmannii* (cv) Valencia peels. An exemplificative chromatogram of the phenolic and betalain profile are shown in Figure 1. Regarding polyphenolic fraction, twelve different compounds (Table 1) were found, four phenolic acids (Ph1 to Ph4), and eight flavonoids (Fv1 to Fv8). The identification of some of these compounds (twelve polyphenolic compounds) were previously described by the authors in a previous work [14] and have also been previously described by other authors in *Opuntia* spp. samples [15,16,17], with the except of peak Fv3 apigenin-*O*-hexoside, which to our knowledge, is the first time reported in *Opuntia* spp. peels. Although, apigenin derivatives have been previously identified in *Opuntia ficus-indica* flowers and cladodes [18,19]. Therefore, the tentative identification of these compounds was assumed taking into account previous findings.

Considering the number of compounds identified in prickly pear peels, flavonoids were the most relevant class of phenolic compounds, although, the most abundant molecule out of the 12 identified phenolic compounds was piscidic acid (Figure 1). This phenolic acid was previously identified in *Opuntia ficus-indica* [14]. Thus, the main family of phenolic acids found in *Opuntia engelmannii* peels were ferulic acid derivatives.

Betalain molecules, concretely betacyanins, display a wide range of hues between reddish-violet color [14,20,21]. In the betalain fractions (Table 1) seven betacyanins were identified (Bc1 to Bc7). Out of the seven betacyanins identified, six common *Opuntia* spp. isomers were found, betanin, isobetanin, gomphrenin I, 17-decarboxy-betanin, betanidin, and isobetanidin (peaks Bc1, Bc2, Bc3, Bc5, Bc6, and Bc7, respectively), having been previously identified by other authors [16,22,23,24]. Thus, (Iso)phyllocactin (peak Bc4) has not been previously identified in *Opuntia* spp., it has been already described in Cactaceae [25,26,27] and in red beets [28,29], as far as we know, this is the first attempt at tentatively identifying this molecule in *O. engelmannii*. 

### 2.2. Model Fitting and Applied Technologies 

To improve the extraction amounts of the different responses, optimization of the extraction processes is required. Thus, in order to find out the relationship between the influence factors and the characteristics of two extraction methodologies, ultrasound and microwave assistance extraction (UAE and MAE, respectively), four independent variables were monitored (Table 2). For the optimization process, 31 samples (runs) were prepared for UAE-RSM and MAE-RSM, based on the central composite design (CCD), which the independent and dependent variables are summarized in Table 2 and response surface methodology plots are shown in Figures S1–S4. The particular interest was to maximize responses, in order to obtain higher amounts of natural colorants “total betacyanins” (Res1), bioactive compounds “total phenolic acids” and “total flavonoids” (Res2 and Res3, respectively), and “extractable solids” (Res4). In the specific case of “antioxidant activity” (Res5) the calculation was performed through the reducing power assay and reported as IC_50_ values, consequently, the main interest was to minimize this value, which represents a higher bioactivity. Although all the color coordinates were measures in every run Table S4, only the parameter the a* of the “color” extracts (Res6) was analyzed in depth; this parameter indicates the qualitative value for the green-red (−a* to +a* respectively) coloration of the diluted extract. In Table 2 description of all calculated coefficients are shown and marked (with a*) where statistical significance (***p***- value = 0.05) were found, these values were also highlighted later in this section. Common statistical and relevant information is also displayed in Table 2, followed by the calculation of the optimum values, which will be explained in-depth in the following subsections.

### 2.3. Effects of Independent Variables on Betacyanins

#### 2.3.1. Maximizing the Betacyanin Content Using UAE 

The experimental conditions and the response values for the UAE-CCD are listed in Table 2, which shows the highest total betacyanin content (197.51 mg/g) with the following condition: time (*t*) 1.5 min, ratio (*S/L*) 5 g/L, methanol concentration (metOH) 50% and ultrasound temperature (*T*) 20 °C (run No. 19). This table also shows the lowest total betacyanin content (72.01 mg/g) with the conditions of (*t*) 1.5 min, (*S/L*) 25 g/L, (metOH) 100 % and (*T*) 20 °C (run No. 22). The response variables were analyzed to fit a regression model. The full quadratic second-order model obtained by multiple regression analysis of the experimental data via RSM was expressed in Equation (1) according to values present in Table 3.

The following equation model (Equation (1)) was used for the prediction of betacyanins content. This equation represents an example of statistically and non-statistical values of second-order polynomial coefficients for betalainic content in the UAE extractions.

(1) $$\begin{array}{c} Res1U=138.6+8.2x_{1}-2.3x_{2}+0.5x_{3}+5.5x_{4}-8.4x_{1}^{2}+0.009x_{2}^{2}-0.02x_{3}^{2}\\ \;\;\;\;-0.08x_{4}^{2}+0.7x_{1}x_{2}-0.08x_{1}x_{3}+0.2x_{1}x_{4}+0.03x_{2}x_{3}-0.09x_{2}x_{4}\\ \;\;\;\;+0.005x_{3}x_{4} \end{array}$$

where Res1U is betacyanin content extracted via UAE, ***x*_1_** is time, ***x*_2_** is the ratio, ***x*_3_** is methanol concentration and ***x*_4_** is ultrasound temperature. In order to simplify data in Table 3, only equation terms are listed, and significant ***p***-values are cited in the text. Then, the second-order model was significant in the linear coefficients ***x*_2_** and ***x*_3_**, and also in the quadratic coefficient $x_{3}^{2}$, respectively. This also included *S/L* ratio (***x*_2_**) as ***p*** = 0.035, ***F*** = 5.28, concentration (***x*_3_**) as ***p*** = 0.0001, ***F*** = 27.35 and quadratic concentration value ($x_{3}^{2}$) as ***p*** = 0.0019, ***F*** = 13.74, which were quite significant with a very small ***p***-value (<0.05), which was determined by the analysis of variance (ANOVA), while the time (***x*_1_**) and temperature (***x*_4_**) had no significant effect on the studied range on betacyanin content (***p*** = 0.7250 and 0.1602, respectively). This indicated that *S/L* ratio and solvent concentration were the most important experimental factors for ultrasound-assisted extraction. Furthermore, the coefficient (R^2^) calculated in the quadratic regression model was higher than 0.77 (Table 3), as well as Durbin–Watson coefficient 2.49, with ***p*** = 0.6713 being greater than ***p*** = 0.05, indicating that the residual distribution does not follow any type of autocorrelation from the regression analysis.

From the ANOVA analysis, in the factorial optimization response section, two of the factors, *S/L* ratio and *metOH* concentration, were the most responsible for the fluctuation on betacyanin content. The low *S/L* ratio was explained by Vinatoru [30], which mention, that increasing solvent volume with respect to the solid sample, provides more surface area for the acoustic wave to form cavitation bubbles and thus increases mass transfer between the solvent and sample. On the other hand, the low methanolic concentration could be explained due to betalains polarity, these water-soluble pigments are normally sequestered in the vacuole of the cell [20,31], so after breaking the cell walls through ultrasonic waves, these molecules are more likely to dissolve in more polar solvents, such as water.

#### 2.3.2. Maximizing the Betacyanin Content Using MAE

**Figure 2:**
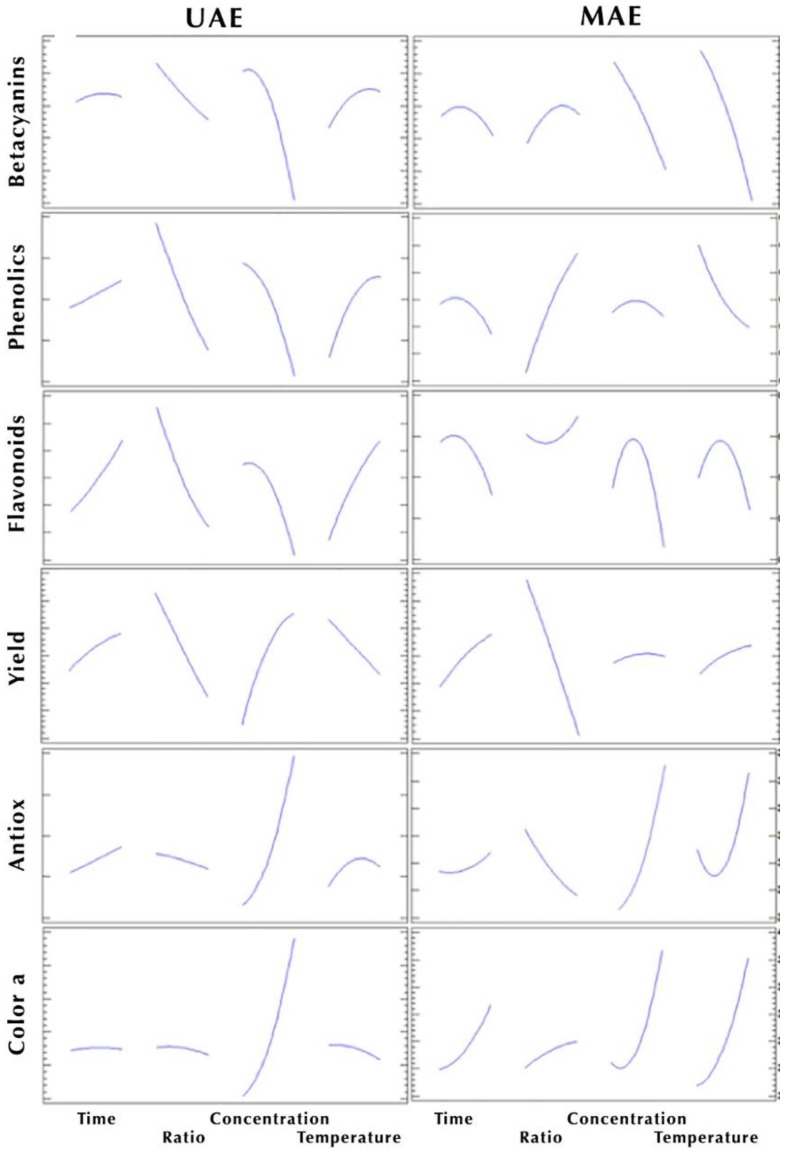
Principal effect graphs obtained for the ultrasound-assisted extraction (UAE) (left column) and microwave-assisted extraction (MAE) (right column), representing responses values, acquire from the independent variables employed on the CCD-RMS (central composite design-response surface methodology).

Optimal extraction conditions for maximum total betacyanins (TB) using MAE were obtained by constructing 3D response surface curves with underlying contour plots (Table S1, supplementary material) and determined by interpolation of experimental values according to Equation (2) (Statistically values of second-order polynomial coefficients for betalainic content in the MAE extractions). MAE process variables significantly affected (***p*** < 0.05) ***b*3** and ***b*4** (***F*** = 33.74 and 65.83, respectively) as a function of methanol concentration and extraction temperature, which showed a marked decrease, with the increase of methanol concentration and temperature as shown in Figure 2. On one hand, as explained before in 3.3.1, the effect of water polarity on betalains extraction has a better performance than the one shown by high methanol concentrations. While on the other hand, according to [32], betalains are susceptible to a wide range of activities, and thermal treatment could lead to wider loss range, from 6%–81% depending on the process applied or temperature used [33,34,35]. Although, Ferreres et al. [36] and Ravichandran et al. [33] report higher betalainic content using MAE, extension of this technique and temperature may also contribute to their degradation.

(2) $$Res1M=-37.7+1.5x_{3}+1.7x_{4}-0.06x_{2}^{2}-0.02x_{3}x_{4}$$

Statistical information of the fitting analysis from Table 3 displays an 89.13 *R^2^* coefficient value from the 31 runs for TB response, which shows a high correlation of the collected data. Table 3 also exposes two different optimization values: 1) The single factorial optimization response, which determines the optimal value of a single response in case of being interested only in maximizing the colorant extraction in a single process, in this case betalainic content of 144.6 mg/g correspondent with factorial values of ***x*_1_** = 8.8 min; ***x*_2_** = 20.3 g/L; ***x*_3_** = 54.8 % of methanol, and ***x*_4_** = 25 °C; and 2) the general optimization response, which determines the optimal value for all the responses together in the same batch, in this case, the max value for betalains it is slightly lower (132.9 mg/g) than the one obtained in the single factorial optimization response, due to maximization of all the other responses, in order to achieve this general optimization, factors are meant to be ***x*_1_** = 12.4 min; ***x*_2_** = 5 g/L; ***x*_3_** = 0 methanol% and ***x*_4_** = 36.6 °C.

### 2.4. Effects of Independent Variables on Phenolic Acids and Flavonoids

#### 2.4.1. Maximizing the Content in Phenolic Acids and Flavonoids Using UAE

From the 31 runs data obtained on Tables S2 and S3 and based on Table 3, total phenolic acids (TPA) and total flavonoids (TF) extracted using UAE were significantly (***p*** ˂ 0.05) affected by the linear effect of *S/L* ratio and *metOH* concentration, while temperature only had a significant effect on TF, and time did not have any effect on both responses. Significant interaction was also observed for *S/L* ratio and *metOH* concentration for both TPA and TF, although, the last-mentioned response, was also significant on ratio–temperature interaction. Remaining factors did not show any significant (***p*** < 0.05) effect on TPA or TF. Removing all non-significant terms, the polynomial Equations (3) and (4) for TPA (Res2U) and TF (Res3U) respectively, are present as following:

(3) $$Res2U=6.263-0.08x_{2}+0.01x_{3}+0.003x_{2}x_{3}$$

(4) $$Res3U=0.434+0.009x_{2}+0.006x_{3}+0.049x_{4}+0.0001x_{2}x_{3}-0.001x_{2}x_{4}$$

Previously, in point 2.3.1 we had proposed a concise response about the sample/solvent ratio in the extraction, which gave us a clue as to why lower ratios rendered higher yields of bioactive compounds. Regarding, the solvent concentration the best yield for these bioactive compounds are achieved by using high concentrations of water rather than methanol (Table 2). A possible explanation could be due to the interaction of both bioactive compounds with water-soluble molecules like carbohydrates or by their carboxylic acid terminations [37]. Most certainly, extraction conditions used, are not strong enough to split the aglycones from the original glycosyl moieties, forcing them to bond more efficiently to low methanolic solvents. Additionally, with the increasing temperature the solubility of both bioactive compounds increases, due to the penetration of solvent into the plant matrix and higher mass transfer rate [38].

#### 2.4.2. Maximizing the Content in Phenolic Acids and Flavonoids Using MAE

From Tables S2 and S3 (supplementary material), piscidic acid is the main extracted molecule, being at least 20-fold higher than the rest of the compounds detected, followed by three most abundant flavonoids, such as apigenin-*O*-hexoside, isorhamnetin-*O*-hexoside-*O*-(di-deoxyhexosyl-hexoside) and isorhamnetin-*O*-(di-deoxyhexosyl-hexoside), respectively. While the other phenolic compounds revealed similar recovery percentages. The data obtained in both TPA (Res2) and TF (Res3) for MAE did not show significant statistical differences for the linear or quadratic terms, and only showed significance (***p*** < 0.05) for the time–concentration interaction for Res2 (TPA). Due to this singular significant coefficient, a polynomial coefficient equation of Res2 and Res3 for the MAE are not shown. The fact that a non-significant result was obtained, could be explained by insufficient time or temperature employed on the MAE system for these particular compounds (phenolic acids and flavonoids).

### 2.5. Effects of Independent Variables on Yield by Applying UAE and MAE

Figure 2 clearly stands out 2 significant factors: (i) *S/L* (***x*_2_**) negatively effects the yield response, meaning, while solid increases in respect to liquid in both type of extractions (UAE and MAE), response yield decreases. On the other hand, in UAE, (ii) *metOH* concentration (***x*_3_**) also played an important role, which augment extractable solid while solvent concentration increases, this effect is confirmed with the statistical significance in Table 3. Higher yields are observed with higher *metOH* concentration, achieving an optimum value for ***x*_3_** in Res4, using a 30.2% of methanol in water. 

The S/L ratio effect on extraction of these phytochemical compounds was mentioned before in Section 2.3.1, standing out that higher S/L ratios provides higher extractions yields. On the other hand, the effect of the solvent concentration on the yield could be explained by the polarity effect, we pointed out that these phytochemicals have a higher extractability with lower methanolic concentration, thus this little increase in methanol could help the extraction of other unidentified polar compounds, and due to its amphiphilic properties, it could also extract some other non-polar [39] molecules, leading to higher yields when applying this type of mixtures. 

### 2.6. Effects of Independent Variables on Antioxidant Activity by Applying UAE and MAE

Our main goal was to minimize the IC_50_ value in Res5 (antioxidant effect). Briefly, lower IC_50_ values means that lower antioxidant molecules are needed to reduce 50% of the reducing power, which means a higher antioxidant activity. Previous antioxidant (hydrophilic and lipophilic) assays were performed on *Opuntia* fruits [14,24], but for sake of convenience, in this study authors worked with reducing power as an indicative antioxidant method.

Betalains and phenolic compounds have shown a wide range of physiological properties, such as antimicrobial, anti-inflammatory, cardioprotective, and antioxidant, among others [14,17,31]. From Table 3 and Figure 2 we were able to identify solvent concentration (***b*3**), as the only linear effect with statistical significance in UAE and MAE for this response criteria. Both extraction methodologies exhibit better antioxidant effect when applying high aqueous concentration, which is in concordance with previously analyzed data, that showed how betalains, phenolic acids and flavonoids concentrations were higher with low methanolic solvents. These results provide a clear synergistic effect between the identified bioactive compounds and the antioxidant effect tested.

### 2.7. Differences Among Employed Technologies: UAE and MAE 

Ultrasound and microwave are both common extraction technologies used for the recovery of bioactive compounds, in bibliography there is a plethora of information about different plants and by-products, mainly oriented to improve specific conditions, although these technologies have different mechanism of action, both these extraction procedures are highly cited methodologies [12]. 

All the phytochemical compounds were identified using both extraction technologies, thus the percentages and the concentrations of these molecules were variable depending on the conditions applied. Considering the optimized conditions in each extraction system (Table 2), all the maximum amounts of the identified bioactive compounds (betalains, phenolic acids, and flavonoids) was achieved when applying the UAE. For instance, regarding betalains, UAE revealed a total content with 22% higher than MAE (197.51 mg/g of UAE vs 151.11 mg/g of MAE); for phenolic acids and flavonoids these differences went up to about 43% (12.99 mg/g of UAE vs 7.46 mg/g of MAE and 2.06 mg/g of UAE vs 1.16 mg/g of MAE, respectively). The differences in terms of specific compounds did not exceed the 2% for betacyanins, 6.1% for flavonoids and 4% for phenolic acids. For instance, in the latter group mentioned, piscidic acid (Ph1) was found in higher concentration in MAE (98.3%) in comparison to UAE (94.3%) among all the molecules distribution, while *cis*-caffeic acid displayed a higher percentage within UAE (4.2%) in comparison to MAE (0.3%), showing different compounds specificity in each treatment. In the antioxidant activity effect (Table 2), although lower IC_50_ response was obtained using the MAE 2.6 mg/mL vs 2.9 from the UAE, the majority of the results obtained for the 31 runs was always better for the UAE extracts. Two possible explanations for this unexpected result could be due to a colorimetric interference while performing the assay, either way, UAE has shown also an overall better antioxidant performance, which is in concordance with the maximum amount of biomolecules obtained for this extraction system. Table 3, also shows the overall optimum responses values of 2.9 mg/mL for UAE and 3.6 mg/mL for MAE, which is in agreement with the above-mentioned. On the other hand, the percentage of yield (Table 2) was in the opposite way, where MAE exhibits the best results in most of the performed runs.

## 3. Material and Methods

### 3.1. Samples Preparation

Cactus pear fruits (*Opuntia engelmannii* cultivar (cv.) Valencia) were manually collected in October 2017 in Valencia, Spain (GPS coordinates: 39°28′34.7” N 0°20′00.4” W). Within 24 h, fruits were washed with distilled water to remove glochids and then further air-dried. Afterwards, all the fruits were peeled and the resulting peel was lyophilized (Telstar Lioalfa 6, Azbil corp., Tokyo, Japan), grounded and sieved using a fine mesh sieve (20 mesh) and stored in a bag under vacuum in a cool and dry place until use.

### 3.2. Experimental Design

In this study, central composite design (CCD) was used for optimization of UAE and MAE of prickly pear peels. The design consisted of 31 randomized runs with seven replicates at the central point. For UAE, the variables in the designed experiment were defined as ***x*_1_**: time (0.5–2.5 min), ***x*_2_**: solid to liquid ratio (5–45 g/L), ***x*_3_**: solvent concentration (0%–100% Methanol/water) and ***x*_4_**: temperature (3–35 °C); while, for MAE they were ***x*_1_**: time (2.5–12.5 min), ***x*_2_**: solid to liquid ratio (5–45 g/L), ***x*_3_**: solvent concentration (0%–100% Methanol/water) and ***x*_4_**: temperature (25–105 °C), and each one was tested at five different levels. The variables ***x*_1_** and ***x*_4_** were selected according to previously executed factorial designs (data not shown).

The dependent variable studied were Response 1: betacyanin content (mg/g of extract), Response 2: zhenolic acids (mg/g of extract), Response 3: flavonoids (mg/g extract), Response 4: extractable solid (%), Response 5: antioxidant activity measured through the reducing power (RP) assay (IC_50_ value in mg/mL) and Response 6: color (a* coordinate).

The experimental data were fitted to the second-order polynomial model (Equation (5)) to obtain the regression coefficients (***b***) using Statgraphics Centurion XVI software (StatPoint Technologies, Inc. Warrenton, VA, USA). The generalized second-order polynomial model used in the response surface analysis was the following:

(5) $$Y\;=\;b_{0}+\;\overset{k}{\underset{i=1}{\sum }}b_{i}x_{i}+\overset{k}{\underset{i=1}{\sum }}b_{ii}x_{i}^{2}+\;\overset{k}{\underset{j=i+1}{\sum }}b_{ij}x_{i}x_{j}$$

where *Y* is the dependent variable (response variable) to be modelled, ***b***_0_ is a constant coefficient (intercept); ***b****_i_*, ***b****_ii_* and ***b****_ij_* are the coefficients of the linear, quadratic, and interactive terms, respectively; *k* is the number of tested variables (*k* = 4); ***x_i_*** and ***x_j_*** are the independent variables. Parametric estimation responses were collected in the form of total batalain content (TB), total flavonoid (TF), total phenolic acids (TPA), RP antioxidant activity, yield, and color parameter.

### 3.3. Extraction Procedure

#### 3.3.1. Ultrasound-Assisted Extraction (UAE)

Ultrasound-assisted extraction of bioactive compounds from *O. engelmannii* was performed using an ultrasonic bath system (ATM40-3LCD, Labbox, Barcelona, Spain) connected to a cooled thermal regulation system, consisting of a cooling bath (Frigiterm-TFT-30, JP Selecta, Barcelona, Spain) joined to an immersion thermostat (Tectron bio, JP Selecta, Barcelona, Spain) and linked throughout water recirculation with a peristaltic pump (Pumpdrive 5006, Heidolph, Schwabach, Germany) in order to precisely control the temperature selected. The necessary amount of lyophilized peel with the designed volume of methanol concentration in the required ratio with a constant volume of 20 mL were placed in an Erlenmeyer flask inside the ultrasonic bath, that was equipped with digital control system for sonication time and frequency (40 ± 2 kHz), and was agitated with an overhead stirrer (RZR 2021, Heidolph, Schwabach, Germany) at the speed of 200 rpm. Prior to extractions, every methanol concentration employed were taken to pH 7 (S20 SevenEasy pH, Mettler Toledo, OH, USA) using McIlvaine buffer solutions. Following the extraction, samples were centrifuged (5804 R centrifuge, Eppendorf, Hamburg, Germany) at 11,000 rpm for 7 min at 10 °C, filtered through Buchner funnel with fritted disc, pore No. 2 (40–90 μm), dried at 40 °C using a vacuum rotary evaporator (Hei-VAP Value, Heidolph, Schwabach, Germany) and lyophilized (Telstar Lioalfa 6, Azbil corp., Tokyo, Japan) to obtain the powder extract. The extracts were stored in sealed vaccum bags in a dry place prior to consequent analysis.

#### 3.3.2. Microwave-Assisted Extraction (MAE)

**S**amples were prepared according to the experimental design, using the same lyophilized peel, solvents (pH 7 adjusted) and extraction volume used in UAE assays. Extractions were performed in a microwave digestion system (Mars 6, CEM, NC, USA) using the MARSxpress plus vessels. The microwave was equipped with a digital control system for irradiation (iWave), time and microwave power (the latter was set at 400 W). After conditions selected were reached, samples were cooled down to room temperature and centrifuged, filtered, dried, lyophilized, and stored as in the UAE extractions.

### 3.4. Responses Analyzed

#### 3.4.1. LC-DAD-ESI/MS Characterization of Extracts

The phytochemical profile of phenolic and betalain compounds were determined by LC-DAD-ESI/MS, using a Dionex Ultimate 3000 UPLC instrument, coupled to a diode-array detector and to a mass spectrometer (MS, Linear Ion Trap LTQ XL) equipped with an ESI source (Thermo Scientific, San Jose, CA, USA).

The phenolic compounds were analyzed using a previously described methodology [40]. Detection was carried out with a DAD (280 nm, 330 nm, and 370 nm as the preferred wavelengths) and in a MS working in negative mode. For betalain compounds a previously described methodology was applied for their identification [14]. Detection was carried out with a DAD (480 and 530 nm as the preferred wavelengths) and in a MS working in positive mode. 

Data acquisition was carried out with Xcalibur^®^ data system (Thermo Finnigan, San Jose, CA, USA) and phenolic and betalain compounds were identified through the available standards and by using previous literature information regarding the fragmentation pattern [41]. The quantification was performed using a 7-level calibration curves (5–200 µg/mL) obtained from commercial standards. For betalains an isolated compound gomphrenin III (isolated from *Gomphrena globosa* L.; y = 14,670x − 19,725, *R^2^* = 0.9997; [16]) was used for their quantification. The results were expressed in mg per g of extract.

#### 3.4.2. Colorimetric Determination 

Color was measured in the re-suspended extracts (methanol: water 80:20 *v/v*, 7.5 mg/mL) using a Minolta spectrophotometer (CM-3600d, Konica Minolta Sensing, Inc., Tokyo, Japan) equipment. Using the reflectance mode and the LAV lens with diffuse illumination 65/10° viewing angle and specular component excluded. The CIE L* values, Cartesian coordinates a* and b* and cylindrical coordinates C* and *h°* were reported throughout Spectra Magic software (version 3.6, CyberChrome Inc., Tokyo, Japan). In this context each coordinate represents, *L*^∗^: lightness, *a*^∗^: chromaticity on a green (−) to red (+) axis, *b*^∗^: chromaticity on a blue (−) to yellow (+) axis, C*: Chroma or relative saturation and *h°*: the hue angle in the CIELab color wheel. 

#### 3.4.3. Extractable Solid (Yield)

Yield parameter was obtained from separating a portion (5 mL) of the filtered extraction liquid obtained from UAE and MAE and placed inside of an oven where both parts of the mixture (methanol/water) solvent were evaporated. Afterwards, the dried sample was cooled down and the residue was calculated by difference.

#### 3.4.4. Antioxidant Activity Evaluation

A stock solution (30 mg/mL) was made from each extract with methanol:water 80:20 v/v and successive dilutions were performed (0.9–15 mg/mL) and submitted to the reducing power (RP) assays to evaluate the antioxidant activity of the samples. The RP assay evaluates the capacity of the extracts to reduce Fe^3+^ to Fe^2+^, measuring the absorbance at 690 nm and was performed according to Melgar et al., [24]. The results were expressed as IC_50_ values (concentration of the sample providing 0.5 of absorbance) for antioxidant activity and Trolox was used as positive control. 

### 3.5. Statistical Analysis

The analysis of variance (ANOVA) was carried out to determine individual linear, quadratic and interaction regression coefficient using Statgraphics Centurion XVI software (StatPoint Technologies, Inc. Warrenton, VA, USA), and the fitness of the polynomial equation to the responses was estimated using the coefficient of determination (*R*^2^). The significance of all the terms of the polynomial equation was analyzed statistically by computing the *F* value at ***p*** < 0.05. Statgraphics software was used to optimize the conditions of extraction throughout response surface methodology (RSM) with their respective 3D graphs.

## 4. Conclusions

The present work has described the use of UAE and MAE techniques in order to extract add-value bioactive compounds from *O. engelmaannii* peels, namely betacyanins, phenolic acids, and flavonoids, as well as their antioxidant activity. In this study, the interaction of four different factors (time, solid to solvent ratio, methanol concentration and temperature) were investigated using a RSM model. In an overall, this study allowed: (i) the identification of 7 different betacyanins, 4 phenolic acids and 8 flavonoids; (ii) the recognition of each independent variable impact on the studied responses, indicating the factorial optimization responses and proposed how to build the equation models for the prediction of the different responses; (iii) a comparison between two extraction techniques, such as UAE and MAE, in order to achieve the best extraction conditions to obtain the highest yields of the detected compounds and bioactivity present in this particular by product.

Finally, a general response optimization of both extractions systems was also performed statistically, involving all the assayed responses at the same time. With a response of 0.985, the optimum values for each factor in the UAE was: ***x*_1_** = 2.5 min, ***x*_2_** = 5 g/L, ***x*_3_** = 34.6% of methanol and ***x*_4_** = 30 °C. In the same manner, with a response of 0.871, the optimum values for each factor in MAE was: ***x*_1_** = 1.4 min, ***x*_2_** = 5 g/L, ***x*_3_** = 0.0% of methanol, and ***x*_4_** = 36.6 °C. These conditions will produce 201.6 and 132.9 mg/g of betalains, 13.9 and 8.0 mg/g of phenolic acids, 2.4 and 1.5 mg/g of flavonoids, with a yield of 71.8% and 79.1% and an IC_50_ value of 2.9 and 3.6 mg/mL, for UAE and MAE respectively. Thus, the present study suggests that the UAE was the best extraction system, in order to maximize recovery of the detected bioactive compounds, which also provide the highest antioxidant activity. 

## Figures and Tables

**Table 1:** Chromatographic and mass characteristic of the tentative identification bioactive compounds in the *Opuntia engelmannii* cv Valencia peels.

Peak	R_t_ (min)	λmax (nm)	[M − H]^−^ (*m/z*)	MS^2^ (*m/z*)	Tentative Identification
Betalains					
Bc1	22.2	534	551	389(100),345(50),150(28)	Betanin ^1^
Bc2	23.7	534	511	389(100), 345(73),150(46)	Isobetanin ^1^
Bc3	23.8	535	551	507(3), 389(38), 345(100), 301(21)	Gomphrenin I ^1^
Bc4	25.2	509	637	551(20),389(54),345(100),150(62)	(Iso)phyllocactin ^1^
Bc5	25.4	505	507	345(100),301(63)	17-Decarboxy-betanin ^1^
Bc6	26.1	523	389	343(97),150(91)	Betanidin ^1^
Bc7	28.0	534	389	389 (100),345(73),150(46)	Isobetanidin ^1^
Phenolic acids					
Ph1	4.2	278	255	193(32),179(7),165(100),149(5)	Piscidic acid ^2^
Ph2	6.5	321	367	193(100),191(12),173(13),149(23)	3-*O*-Feruloylquinic acid ^3^
Ph3	7.7	285	179	161(100),143(79),119(32)	*cis* Caffeic acid ^4^
Ph4	8.8	283	355	193(100)	Ferulic acid hexoside ^3^
Flavonoids					
Fv1	10.5	331	931	769(31),315(100)	Isorhamnetin-*O*-hexoside-*O*-(di-deoxyhexosyl-hexoside) ^5^
Fv2	12.3	338	785	315(100)	Isorhamnetin-*O*-hexoside-(deoxyhexosyl-hexoside) ^5^
Fv3	16.2	345	431	269(100)	Apigenin-*O*-hexoside ^6^
Fv4	16.6	346	931	769(43),315(100)	Isorhamnetin-dirutinoside ^5^
Fv5	16.9	332	931	769(27),315(100)	Isorhamnetin-dirutinoside ^5^
Fv6	17.3	331	769	315(100)	Isorhamnetin-*O*-(di-deoxyhexosyl-hexoside) ^5^
Fv7	18.2	325	785	315(100)	Isorhamnetin-*O*-hexoside-(deoxyhexosyl-hexoside) ^5^
Fv8	19.0	337	623	315(100)	Isorhamentin-*O*-(deoxyhexosyl-hexoside) ^5^

Calibration curves used. 1—gomphrenin III (y = 14670x − 19725); 2—*p*-hydroxybenzoic acid (y = 208604x + 173056); 3—ferulic acid (y = 633126x − 185462); 4—caffeic acid (y = 388345x + 406369); 5—quercetin-3-*O*-glucoside (y = 34843x − 160173); 6—apigenin-7-*O*-glucoside (y = 10683x − 45794).

**Table 2:** Central composite design and experimental data for 4-level-4-factor response surface analysis.

Run	*X*_1_		*X*_2_	*X*_3_	*X*_4_		Res1		Res2		Res3		Res4		Res5		Res6	
	UAE	MAE	UAE/MAE	UAE/MAE	UAE	MAE	UAE	MAE	UAE	MAE	UAE	MAE	UAE	MAE	UAE	MAE	UAE	MAE
1	1	5	15	25	12.5	45.0	182	112	8.9	6.4	1.13	0.94	62	66	3.1	4.0	83.2	75.0
2	2	10	15	25	12.5	45.0	149	140	7.3	7.0	0.95	1.16	64	67	3.4	3.8	83.6	74.4
3	1	5	35	25	12.5	45.0	134	112	6.4	5.7	0.77	0.80	60	62	3.4	4.2	83.3	74.3
4	2	10	35	25	12.5	45.0	153	129	7.3	6.6	0.88	0.76	61	51	3.4	3.4	83.4	77.8
5	1	5	15	75	12.5	45.0	109	108	6.7	5.8	0.73	0.72	66	73	3.6	4.5	83.1	72.3
6	2	10	15	75	12.5	45.0	113	113	6.4	6.2	0.96	1.09	66	71	3.6	3.7	85.8	72.6
7	1	5	35	75	12.5	45.0	124	141	7.0	7.5	0.96	1.13	66	40	3.1	3.3	86.6	78.0
8	2	10	35	75	12.5	45.0	128	120	7.0	6.4	0.88	0.89	64	65	3.7	3.5	86.3	77.3
9	1	5	15	25	27.5	85.0	170	83	8.6	5.6	1.14	0.90	63	67	3.1	3.6	83.1	73.8
10	2	10	15	25	27.5	85.0	190	81	13	5.6	2.06	0.88	63	89	3.0	4.1	83.0	73.5
11	1	5	35	25	27.5	85.0	133	116	6.6	6.2	0.79	1.02	44	65	3.4	3.6	83.8	75.2
12	2	10	35	25	27.5	85.0	139	108	6.8	6.7	0.89	0.86	57	55	3.4	3.7	83.3	76.3
13	1	5	15	75	27.5	85.0	143	54	7.7	7.2	1.13	1.03	65	70	3.4	2.6	85.7	59.3
14	2	10	15	75	27.5	85.0	125	25	7.0	4.0	0.96	0.47	66	66	3.9	3.9	85.4	74.9
15	1	5	35	75	27.5	85.0	116	66	6.5	6.3	0.83	0.93	64	67	3.6	3.4	85.3	76.4
16	2.5	10	35	75	27.5	85.0	119	60	6.8	6.7	0.95	0.91	63	65	3.7	3.9	86.4	79.0
17	0.5	2.5	25	50	20	65.0	159	107	8.4	6.2	0.96	0.87	64	56	3.4	3.6	85.2	74.0
18	2.5	12.5	25	50	20	65.0	174	77	8.4	6.2	1.21	0.94	65	68	3.1	3.7	84.7	75.5
19	1.5	7.5	5	50	20	65.0	198	97	9.6	6.0	1.28	0.99	68	72	3.5	4.1	84.8	72.9
20	1.5	7.5	45	50	20	65.0	160	80	7.8	6.6	1.02	0.99	63	53	3.0	3.6	84.8	75.3
21	1.5	7.5	25	0	20	65.0	175	155	8.2	6.4	1.03	0.86	62	59	3.1	3.3	83.2	76.1
22	1.5	7.5	25	100	20	65.0	72	53	6.5	6.1	0.64	0.76	62	65	4.0	3.8	76.3	74.0
23	1.5	7.5	25	50	5	25.0	134	135	6.7	6.8	0.81	0.78	65	69	3.0	3.1	84.9	77.7
24	1.5	7.5	25	50	35	105	179	49	8.3	6.6	1.15	0.93	65	56	3.2	5.7	84.7	73.1
25	1.5	7.5	25	50	20	65.0	143	185	7.4	6.7	0.99	1.04	63	59	3.5	3.5	84.5	76.7
26	1.5	7.5	25	50	20	65.0	184	115	9.2	6.0	1.21	0.87	64	69	3.4	3.7	84.5	73.5
27	1.5	7.5	25	50	20	65.0	135	112	6.6	5.9	0.96	0.93	64	68	3.5	3.6	86.5	73.5
28	1.5	7.5	25	50	20	65.0	187	127	9.0	6.2	1.20	0.86	64	68	3.4	3.5	85	74.1
29	1.5	7.5	25	50	20	65.0	161	126	7.7	6.2	0.98	0.90	64	67	3.3	3.5	85.2	74.0
30	1.5	7.5	25	50	20	65.0	169	115	8.6	6.8	0.95	1.09	64	65	2.9	3.3	84.8	73.3
31	1.5	7.5	25	50	20	65.0	165	112	8.0	7.0	1.03	1.08	65	64	3.4	3.3	84.6	74.1

*X*_1_: Time (min), *X*_2_: Ratio (g/L), *X*_3_: Concentration (% methanol), *X*_4_: Temperature (°C), and Res1: Total betacyanins (mg/g), Res2: Total phenolic acids (mg/g), Res3: Total flavonoids (mg/g), Res4: Extractable solid (mg/g), Res5: Antioxidant activity (mg/mL), Res6: Color (a* coordinates).

**Table 3:** Statistical analysis (ANOVA) of the central composite design, including response terms for building the predictive models and optimal response values for the parametric response criteria.

		UAE Extraction						MAE Extraction					
		Res1U	Res2U	Res3U	Res4U	Res5U	Res6U	Res1M	Res2M	Res3M	Res4M	Res5M	Res6M
**Intercept**	**b0**	138.65	6.26	0.43	67.40	3.15	45.16	−37.68	5.74	0.03	61.48	9.38	65.60
**Linear**	**b1**	8.21	−0.23	0.12	2.21	−0.04	0.02	23.64	0.50	0.16	2.66	−0.42	* −2.814
	**b2**	* −2.278	* −0.085	* −0.009	* −0.304	<0.01	−0.04	1.98	−0.11	−0.02	* −0.720	−0.05	0.21
	**b3**	* 0.501	* 0.017	* 0.006	* 0.067	* −0.008	* −0.069	* 1.514	0.03	0.01	−0.01	−0.01	* −0.542
	**b4**	5.60	0.32	* 0.049	−0.19	0.01	0.06	* 1.739	−0.02	0.01	0.19	−0.11	* −0.515
**Quadratic**	**b11**	−8.47	0.01	0.03	−0.76	<0.001	−0.27	−0.88	−0.01	<0.001	−0.07	<0.001	0.08
	**b22**	0.01	<0.001	<0.001	<0.001	<0.001	<0.001	* −0.064	<0.001	<0.001	<0.001	<0.001	<0.001
	**b33**	* −0.021	<0.001	<0.001	<0.001	<0.001	*0.001	<0.001	<0.001	<0.001	<0.001	<0.001	* 0.003
	**b44**	−0.08	<0.001	<0.001	<0.001	<0.001	<0.001	−0.01	<0.001	<0.001	<0.001	* 0.001	* 0.003
**Interaction**	**b12**	0.76	<0.001	−0.01	0.09	<0.001	0.02	−0.06	0.01	<0.001	−0.03	<0.001	−0.01
	**b13**	−0.09	−0.02	<0.001	−0.08	<0.001	<0.001	−0.08	* −0.005	<0.001	0.02	<0.001	0.02
	**b14**	0.27	0.08	0.01	0.16	−0.01	<0.001	−0.09	<0.001	<0.001	−0.01	0.01	0.02
	**b23**	0.03	* 0.003	* <0.001	0.01	<0.001	<0.001	0.01	<0.001	<0.001	<0.001	<0.001	<0.001
	**b24**	−0.09	−0.01	* −0.001	−0.02	<0.001	<0.001	0.02	<0.001	<0.001	0.01	<0.001	<0.001
	**b34**	0.01	<0.001	<0.01	0.01	<0.001	<0.001	* −0.021	<0.001	<0.001	<0.001	<0.001	* 0.003
**Statistical information of the fitting analysis**													
**Observations**		31	31	31	31	31	31	31	31	31	31	31	31
**R^2^**		77.76	70.70	75.51	71.03	60.11	93.41	89.13	53.65	54.06	59.93	65.68	80.34
**R^2^adj**		58.31	45.07	54.08	45.68	25.21	87.64	79.61	13.09	13.86	24.87	35.66	63.15
**MSE**		18.65	0.99	0.16	2.99	0.23	0.85	13.99	0.58	0.13	8.54	0.52	2.65
**RMSE**		4.32	0.99	0.40	1.73	0.48	0.92	3.74	0.76	0.36	2.92	0.72	1.63
**MAPE**		10.42	0.55	0.09	1.45	0.13	0.46	8.29	0.34	0.08	4.72	0.27	1.43
**DW**		2.19	1.76	1.78	* 1.01	1.48	1.62	1.94	1.70	1.53	2.05	2.19	1.68
**Factorial Optimization Response**													
		**max**	**max**	**max**	**max**	**min**	**max**	**max**	**max**	**max**	**max**	**min**	**max**
**Optimum value**		227.6	17.8	3.1	73.1	2.3	87.1	144.6	8.6	1.5	84.0	1.8	73.7
***x*_1_**		1.2	2.5	2.5	2.5	2.2	2.1	8.8	2.5	12.5	11.9	12.5	12.4
***x*_2_**		5.0	5.0	5.0	5.3	5.0	44.3	20.3	34.0	5.0	5.0	44.3	16.2
***x*_3_**		17.7	0.1	0.0	30.2	0.0	56.3	54.8	100.0	25.1	46.8	0.0	100.0
***x*_4_**		33.9	34.1	33.8	34.7	35.0	5.0	25.0	103.6	25.0	25.0	25.0	105.0
**General Optimization**													
		**max**	**max**	**max**	**max**	**min**	**max**	**max**	**max**	**max**	**max**	**min**	**max**
**Optimum response**		201.6	13.9	2.4	71.8	2.9	85.7	132.9	8.0	1.5	79.1	3.6	78.7
**Deseability**		0.985						0.871					
**Optimum value**				***x*_1_** = 2.5	**x_2_** = 5	**x_3_** = 34.6	**x_4_** = 30.0		**x_1_** = 12.4	**x_2_** = 5.0	**x_3_** = 0.0	***x*_4_** = 36.6	

Numbers in linear quadratic and interaction marked with (*) means statistically significant based on ***F*** and ***p***-values cited in the correspondent subsections. Terms included: R2: regression coefficients; R2adj: adjusted regression coefficients; MSE: Minimum Square Error; RMSE Root Minimum Square Error; MAPE: Mean Absolute Percentage Error. DW: Durbin–Watson statistic. Responses are summarized as Res1: Total betacyanins (mg/g). Res2: Total phenolic acids (mg/g). Res3: Total flavonoids (mg/g). Res4: Extractable solid (mg/g). Res5: Antioxidant activity (mg/mL). Res6: Color (a* coordinates). Fixed variables are summarized as ***x*_1_** = time (min); ***x*_2_** = ratio (g/L); ***x*_3_** = methanol concentration (%); ***x*_4_** = temperature (°C).

## Supplementary Materials

The following are available online at. Table S1: Betacyanins (mg/g of extract) identified in every single run and the total betacyanin content., Table S2: Phenolic acids (mg/g of extract) identified in every single run and the total phenolic acid content., Table S3: Flavonoids (mg/g of extract) identified in every single run and the total flavonoid content., Figures S1–S4: Total betacyanin content (S1), total phenolic content (S2), total flavonoid content (S3), antioxidant activity (S4) 3d response surface plots of UAE and MAE for the parametric responses., Table S4. Color coordinates acquired in the 31 runs for UAE and MAE. Terms used include: L*: lightness; a*: green-red color component; b*: blue-yellow color component and C: Chroma.

## References

[1] GVR Natural Antioxidants Market Analysis By Product (Vitamin C, Vitamin E, Polyphenols, Carotenoids) And Segment Forecasts To 2022. https://www.grandviewresearch.com/industry-analysis/natural-antioxidants-market.

[2] Gebhardt D. The Economics of Natural Color Pigments. https://sensientfoodcolors.com/en-us/research-development/economics-natural-color-pigments/.

[3] do Prado D.Z., Capoville B.L., Delgado C.H.O., Heliodoro J.C.A., Pivetta M.R., Pereira M.S., Zanutto M.R., Novelli P.K., Francisco V.C.B., Fleuri L.F. (2018). Nutraceutical food: Composition, biosynthesis, therapeutic properties, and applications. Altern. Replace. Foods.

[4] IFIC FDA Overview of Food Ingredients, Additives and Colors. https://www.fda.gov/food/ingredientspackaginglabeling/foodadditivesingredients/ucm094211.htm.

[5] Aruwa C.E., Amoo S.O., Kudanga T. (2018). Opuntia (Cactaceae) plant compounds, biological activities and prospects—A comprehensive review. Food Res. Int..

[6] Cardoso-Ugarte G.A., Sosa-Morales M.E., Ballard T., Liceaga A., San Martín-González M.F. (2014). Microwave-assisted extraction of betalains from red beet (Beta vulgaris). LWT-Food Sci. Technol..

[7] Garcia-Castello E.M., Rodriguez-Lopez A.D., Mayor L., Ballesteros R., Conidi C., Cassano A. (2015). Optimization of conventional and ultrasound assisted extraction of flavonoids from grapefruit (Citrus paradisi L.) solid wastes. LWT-Food Sci. Technol..

[8] Laqui-Vilca C., Aguilar-Tuesta S., Mamani-Navarro W., Montaño-Bustamante J., Condezo-Hoyos L. (2018). Ultrasound-assisted optimal extraction and thermal stability of betalains from colored quinoa (Chenopodium quinoa Willd) hulls. Ind. Crops Prod..

[9] Thirugnanasambandham K., Sivakumar V. (2017). Microwave assisted extraction process of betalain from dragon fruit and its antioxidant activities. J. Saudi Soc. Agric. Sci..

[10] Chemat F., Vian M.A., Cravotto G. (2012). Green extraction of natural products: Concept and principles. Int. J. Mol. Sci..

[11] Barba F.J., Puértolas E., Brnčić M., Panchev I.N., Dimitrov D.A., Athès-Dutour V., Moussa M., Souchon I. (2015). Chapter 11—Emerging extraction. Food Waste Recover..

[12] Mandal S.C., Mandal V., Das A.K. (2015). Classification of Extraction Methods. Essentials of Botanical Extraction.

[13] Leardi R. (2013). Experimental Design.

[14] Melgar B., Dias M.I., Ciric A., Sokovic M., Garcia-Castello E.M., Rodriguez-Lopez A.D., Barros L., Ferreira I. (2017). By-product recovery of Opuntia spp. peels: Betalainic and phenolic profiles and bioactive properties. Ind. Crops Prod..

[15] Chougui N., Djerroud N., Naraoui F., Hadjal S., Aliane K., Zeroual B., Larbat R. (2015). Physicochemical properties and storage stability of margarine containing Opuntia ficus-indica peel extract as antioxidant. Food Chem..

[16] Mena P., Tassotti M., Andreu L., Nuncio-Jáuregui N., Legua P., Del Rio D., Hernández F. (2018). Phytochemical characterization of different prickly pear (Opuntia ficus-indica (L.) Mill.) cultivars and botanical parts: UHPLC-ESI-MSnmetabolomics profiles and their chemometric analysis. Food Res. Int..

[17] Yeddes N., Chérif J., Guyot S., Sotin H., Ayadi M. (2013). Comparative Study of Antioxidant Power, Polyphenols, Flavonoids and Betacyanins of the Peel and Pulp of Three Tunisian Opuntia Forms. Antioxidants.

[18] Allai L., Druart X., Öztürk M., BenMoula A., Nasser B., El Amiri B. (2016). Protective effects of Opuntia ficus-indica extract on ram sperm quality, lipid peroxidation and DNA fragmentation during liquid storage. Anim. Reprod. Sci..

[19] Ammar I., Ben Salem M., Harrabi B., Mzid M., Bardaa S., Sahnoun Z., Attia H., Ennouri M. (2018). Anti-inflammatory activity and phenolic composition of prickly pear (Opuntia ficus-indica) flowers. Ind. Crops Prod..

[20] Esquivel P., Carle R., Schweiggert R.M. (2016). Handbook on Natural Pigments in Food and Beverages Industrial Applications for Improving Food Colorl.

[21] Szot D., Skopińska A., Wybraniec S. (2015). Decomposition of 17-decarboxy-betanin in selected aqueous-organic solutions induced by Cu (II) cations. PhD Interdiscip. J. Politech. Gdansk Univ..

[22] Betancourt C., Cejudo-Bastante M.J., Heredia F.J., Hurtado N. (2017). Pigment composition and antioxidant capacity of betacyanins and betaxanthins fractions of Opuntia dillenii (Ker Gawl) Haw cactus fruit. Food Res. Int..

[23] Mata A., Ferreira J.P., Semedo C., Serra T., Duarte C.M.M., Bronze M.R. (2016). Contribution to the characterization of Opuntia spp. juices by LC–DAD–ESI-MS/MS. Food Chem..

[24] Melgar B., Pereira E., Oliveira M.B.P.P., Garcia-Castello E.M., Rodriguez-Lopez A.D., Sokovic M., Barros L., Ferreira I.C.F.R. (2017). Extensive profiling of three varieties of Opuntia spp. fruit for innovative food ingredients. Food Res. Int..

[25] Fathordoobady F., Mirhosseini H., Selamat J., Manap M.Y.A. (2016). Effect of solvent type and ratio on betacyanins and antioxidant activity of extracts from Hylocereus polyrhizus flesh and peel by supercritical fluid extraction and solvent extraction. Food Chem..

[26] García-Cruz L., Dueñas M., Santos-Buelgas C., Valle-Guadarrama S., Salinas-Moreno Y. (2017). Betalains and phenolic compounds profiling and antioxidant capacity of pitaya (Stenocereus spp.) fruit from two species (S. Pruinosus and S. stellatus). Food Chem..

[27] Herbach K.M., Stintzing F.C., Carle R. (2005). Identification of heat-induced degradation products from purified betanin, phyllocactin and hylocerenin by high-performance liquid chromatography/ electrospray ionization mass spectrometry. Rapid Commun. Mass Spectrom..

[28] Spórna-Kucab A., Ignatova S., Garrard I., Wybraniec S. (2013). Versatile solvent systems for the separation of betalains from processed Beta vulgaris L. juice using counter-current chromatography. J. Chromatogr. B Anal. Technol. Biomed. Life Sci..

[29] Wybraniec S., Starzak K., Szneler E., Pietrzkowski Z. (2016). Separation of chlorinated diastereomers of decarboxy-betacyanins in myeloperoxidase catalyzed chlorinated Beta vulgaris L. extract. J. Chromatogr. B Anal. Technol. Biomed. Life Sci..

[30] Vinatoru M. (2001). An overview of the ultrasonically assisted extraction of bioactive principles from herbs. Ultrason. Sonochem..

[31] Strack D., Vogt T., Schliemann W. (2003). Recent advances in betalain research. Phytochemistry.

[32] Sawicki T., Wiczkowski W. (2018). The effects of boiling and fermentation on betalain profiles and antioxidant capacities of red beetroot products. Food Chem..

[33] Ravichandran K., Saw N.M.M.T., Mohdaly A.A.A., Gabr A.M.M., Kastell A., Riedel H., Cai Z., Knorr D., Smetanska I. (2013). Impact of processing of red beet on betalain content and antioxidant activity. Food Res. Int..

[34] Paciulli M., Medina-Meza I.G., Chiavaro E., Barbosa-Cánovas G.V. (2016). Impact of thermal and high pressure processing on quality parameters of beetroot (Beta vulgaris L.). LWT-Food Sci. Technol..

[35] Guldiken B., Toydemir G., Nur Memis K., Okur S., Boyacioglu D., Capanoglu E. (2016). Home-processed red beetroot (Beta vulgaris L.) products: Changes in antioxidant properties and bioaccessibility. Int. J. Mol. Sci..

[36] Ferreres F., Grosso C., Gil-Izquierdo A., Valentão P., Mota A.T., Andrade P.B. (2017). Optimization of the recovery of high-value compounds from pitaya fruit by-products using microwave-assisted extraction. Food Chem..

[37] Belščak-Cvitanović A., Durgo K., Huđek A., Bačun-Družina V., Komes D., Galanakis C.M. (2018). Metabolism and Health Effects of Polyphenols. Polyphenols: Properties, Recovery, and Applications.

[38] Al-Farsi M.A., Lee C.Y. (2008). Optimization of phenolics and dietary fibre extraction from date seeds. Food Chem..

[39] Primorac T., Požar M., Sokolić F., Zoranić L., Urbic T. (2018). A simple two dimensional model of methanol. J. Mol. Liq..

[40] Bessada S.M.F., Barreira J.C.M., Barros L., Ferreira I.C.F.R., Oliveira M.B.P.P. (2016). Phenolic profile and antioxidant activity of Coleostephus myconis (L.) Rchb.f.: An underexploited and highly disseminated species. Ind. Crops Prod..

[41] Roriz C.L., Barros L., Prieto M.A., Morales P., Ferreira I.C.F.R. (2017). Floral parts of Gomphrena globosa L. as a novel alternative source of betacyanins: Optimization of the extraction using response surface methodology. Food Chem..

