# Supplementary material for: Ultrasound and Microwave Assisted Extraction of Opuntia Fruit Peels Biocompounds: Optimization and Comparison Using RSM-CCD
Source: Molecules. 2019 Oct 8;24(19):3618. doi: 10.3390/molecules24193618 (PMC6804160; doi:10.3390/molecules24193618)

Appendix A

Table S1. Betacyanins (mg/g of extract) identified in every single run and the total betacyanin content.

Run	Peak 1 Betanin		Peak 2 Isobetanin		Peak 3 Gomphrenin I		Peak 4 (Iso)phyllocactin		Peak 5 17 decarboxy-betanin		Peak 6 Betanidin		Peak 7 Isobetanidin		Total betacyanins mg/g	
	UAE	MAE	UAE	MAE	UAE	MAE	UAE	MAE	UAE	MAE	UAE	MAE	UAE	MAE	UAE	MAE
1	144.0	90.4	21.6	12.6	4.8	1.7	2.2	0.4	2.0	0.6	3.3	1.2	3.7	5.4	181.5	112.3
2	118.7	111.9	18.0	16.0	4.1	3.0	1.7	1.2	1.6	0.9	2.3	1.5	2.5	5.7	148.8	140.1
3	107.6	95.2	16.2	13.0	3.3	1.8	1.6	1.0	1.3	0.7	1.8	0.7	2.6	0.0	134.3	112.5
4	123.7	104.8	17.7	15.1	3.9	2.3	1.9	0.6	1.6	0.2	2.3	2.0	1.9	4.2	152.9	129.3
5	91.0	91.5	11.1	12.2	2.8	2.0	1.0	1.0	0.9	0.8	0.9	0.3	1.5	0.0	109.3	107.9
6	92.2	91.7	12.4	14.2	2.9	2.2	1.4	0.3	1.0	0.6	1.2	0.9	1.3	3.5	112.5	113.5
7	100.4	117.1	13.4	17.2	3.2	2.5	1.4	0.4	1.1	0.5	1.3	0.3	2.7	3.3	123.6	141.3
8	103.6	96.1	13.8	15.6	3.2	2.1	1.5	0.9	1.1	0.6	1.8	2.0	2.8	2.5	127.8	119.7
9	135.2	67.6	20.8	10.0	4.5	1.0	1.8	0.2	1.8	0.4	2.8	0.1	3.5	3.4	170.3	82.7
10	150.2	65.3	22.7	9.6	5.2	1.0	2.2	0.2	2.3	0.4	3.2	0.8	3.7	3.1	189.5	80.5
11	107.5	92.4	16.6	16.4	3.3	1.6	1.2	0.1	1.2	0.6	1.8	1.0	1.6	4.1	133.2	116.4
12	111.5	83.0	16.5	14.4	3.7	1.7	1.5	0.2	1.4	0.1	2.2	3.7	1.7	4.4	138.6	107.6
13	116.1	43.1	16.0	7.5	4.1	1.0	1.8	0.2	1.5	0.1	2.1	0.1	1.8	1.5	143.2	53.5
14	102.6	18.8	13.4	3.3	3.3	0.3	1.2	0.1	1.1	0.2	1.3	0.3	1.8	2.0	124.7	25.1
15	94.8	52.1	12.7	10.6	2.9	1.2	1.1	0.7	0.9	0.4	1.6	1.2	1.6	0.0	115.7	66.3
16	97.1	44.2	13.4	10.0	3.0	1.2	1.4	0.2	1.0	0.2	1.1	0.7	2.4	3.1	119.3	59.6
17	126.3	88.7	19.1	14.8	3.9	1.8	1.8	0.5	1.3	0.2	2.2	0.6	4.4	0.0	159.1	106.6
18	136.3	60.9	21.3	10.8	4.6	1.5	2.1	0.1	1.8	0.2	3.3	2.3	4.5	1.3	173.9	77.1
19	156.1	78.6	23.6	11.5	5.1	1.4	2.3	0.4	2.0	0.4	3.7	0.5	4.6	4.0	197.5	96.8

20	126.6	55.9	18.8	16.5	4.4	2.2	2.3	0.9	1.6	0.7	2.6	1.5	3.5	2.2	159.7	79.8
21	139.0	127.0	21.4	18.2	4.6	3.4	2.1	1.2	2.4	0.9	2.8	1.3	2.2	3.2	174.5	155.1
22	58.6	16.7	7.2	30.8	1.8	5.0	0.8	0.1	0.6	0.6	0.9	0.0	2.1	0.0	72.0	53.2
23	108.0	113.3	15.2	15.8	3.4	2.5	1.7	1.6	1.3	1.2	2.0	0.8	2.1	0.0	133.8	135.1
24	140.6	25.8	21.8	16.5	5.1	4.8	2.5	0.9	1.7	0.3	3.4	1.2	4.1	0.0	179.2	49.4
25	116.3	92.1	16.1	12.5	3.7	1.5	1.4	0.5	1.2	0.2	1.7	0.7	2.6	0.0	143.0	107.6
26	144.9	95.1	23.2	14.9	5.3	2.1	2.3	1.3	1.9	0.9	3.1	0.5	3.6	0.0	184.3	114.8
27	106.8	93.0	16.9	14.4	3.6	1.8	1.5	0.8	1.3	0.8	2.2	1.0	2.3	0.0	134.7	111.8
28	149.9	102.8	22.8	16.1	5.1	2.4	2.4	0.2	1.8	0.7	2.8	0.8	2.7	4.1	187.4	127.1
29	128.8	101.6	19.7	15.9	4.3	1.8	2.0	0.3	1.6	0.7	2.2	1.3	2.6	4.6	161.2	126.3
30	134.7	94.1	19.4	14.4	4.8	1.6	2.3	0.3	1.7	0.5	2.5	0.3	3.6	3.4	169.0	114.7
31	131.6	90.4	19.6	12.6	4.4	1.7	2.1	0.4	1.7	0.6	2.5	1.2	3.2	5.4	165.0	112.3

Table S2. Phenolic acids (mg/g of extract) identified in every single run and the total phenolic acid content.

Run	Peak 1<br>Piscidic acid		Peak 2<br>3-*O*-Feruloylquinic acid		Peak 3<br>*cis*-Caffeic acid		Peak 4<br>Ferulic acid hexoside		Total phenolic acids<br>mg/g	
	UAE	MAE	UAE	MAE	UAE	MAE	UAE	MAE	UAE	MAE
1	8.46	6.30	n/d	0.03	0.38	0.02	0.07	0.07	8.91	6.41
2	6.85	6.89	n/d	0.02	0.36	0.02	0.06	0.08	7.26	7.01
3	6.00	5.57	0.08	0.02	0.25	0.01	0.05	0.07	6.39	5.66
4	6.86	6.51	n/d	0.02	0.37	0.03	0.06	0.07	7.29	6.62
5	6.34	5.71	0.09	0.02	0.21	0.00	0.05	0.06	6.69	5.80
6	6.03	6.12	0.08	0.02	0.24	0.01	0.05	0.08	6.40	6.23
7	6.59	7.32	n/d	0.03	0.34	0.02	0.06	0.09	6.98	7.46
8	6.62	6.33	n/d	0.02	0.35	0.01	0.06	0.06	7.04	6.42
9	8.11	5.52	n/d	0.01	0.43	0.01	0.07	0.07	8.61	5.60
10	12.13	5.50	0.71	0.01	0.06	0.01	0.09	0.07	12.99	5.59
11	6.23	6.07	0.07	0.03	0.24	0.02	0.05	0.08	6.60	6.20
12	6.44	6.54	n/d	0.01	0.32	0.04	0.05	0.07	6.81	6.65
13	7.28	7.14	0.00	0.01	0.36	0.02	0.07	0.06	7.71	7.23
14	6.60	3.95	n/d	0.01	0.34	0.01	0.06	0.04	7.00	4.01
15	6.13	6.22	0.09	0.03	0.25	0.00	0.06	0.07	6.52	6.32
16	6.33	6.59	n/d	0.04	0.36	0.02	0.06	0.07	6.75	6.73
17	7.93	6.08	0.10	0.02	0.28	0.01	0.06	0.06	8.37	6.18
18	7.92	6.12	n/d	0.02	0.40	0.01	0.07	0.07	8.39	6.22
19	9.05	5.94	n/d	0.01	0.45	0.01	0.08	0.07	9.58	6.03
20	7.33	6.48	n/d	0.01	0.35	0.02	0.06	0.07	7.75	6.57

21	7.78	6.34	n/d	0.01	0.38	0.03	0.06	0.06	8.22	6.43
22	6.21	5.99	n/d	0.04	0.28	0.01	0.05	0.06	6.53	6.10
23	6.28	6.70	0.12	0.01	0.21	0.01	0.05	0.06	6.66	6.78
24	7.83	6.48	n/d	0.02	0.41	0.02	0.06	0.07	8.31	6.60
25	7.00	6.61	0.10	0.04	0.28	0.02	0.06	0.07	7.44	6.74
26	8.61	5.93	n/d	0.00	0.49	0.01	0.07	0.07	9.17	6.02
27	6.17	5.76	n/d	0.02	0.34	0.02	0.05	0.08	6.56	5.87
28	8.41	6.07	0.19	0.03	0.30	0.04	0.07	0.06	8.97	6.20
29	7.19	6.06	0.12	0.02	0.29	0.01	0.06	0.08	7.66	6.18
30	8.00	6.68	0.13	0.02	0.35	0.02	0.07	0.09	8.55	6.81
31	7.57	6.84	n/d	0.02	0.35	0.01	0.06	0.08	7.98	6.95

Table S3. Flavonoids (mg/g of extract) identified in every single run and the total flavonoid content.

Run	Peak 1		Peak 2		Peak 3		Peak 4		Peak 5		Peak 6		Peak 7		Peak 8		Total flavonoids	
	Isorhamnetin-*O*-hexoside-*O*-(di-deoxyhexosyl-hexoside)		Isorhamnetin-*O*-hexoside-(deoxyhexosyl-hexoside)		Apigenin-*O*-hexoside		Isorhamentin-dirutinoside		Isorhamentin-dirutinoside		Isorhamnetin-*O*-(di-deoxyhexosyl-hexoside)		Isorhamnetin-*O*-hexosyl-(deoxyhexosyl-hexoside)		Isorhamentin-O-(deoxyhexosyl-hexoside)		mg/g	
	UAE	MAE	UAE	MAE	UAE	MAE	UAE	MAE	UAE	MAE	UAE	MAE	UAE	MAE	UAE	MAE	UAE	MAE
1	0.38	0.23	0.09	0.06	0.31	0.29	0.11	0.08	0.03	0.03	0.13	0.14	0.08	0.10	0.00	0.02	1.13	0.94
2	0.34	0.29	0.03	0.10	0.30	0.35	0.09	0.09	0.02	0.03	0.11	0.18	0.06	0.09	0.01	0.03	0.95	1.16
3	0.22	0.22	0.04	0.04	0.25	0.25	0.08	0.06	0.02	0.02	0.10	0.13	0.06	0.06	0.00	0.01	0.77	0.80
4	0.31	0.20	0.03	0.03	0.24	0.26	0.09	0.07	0.02	0.03	0.10	0.11	0.06	0.06	0.02	0.01	0.88	0.76
5	0.25	0.23	0.03	0.07	0.20	0.18	0.09	0.02	0.02	0.02	0.08	0.13	0.05	0.06	0.01	0.03	0.73	0.72
6	0.30	0.24	0.05	0.10	0.28	0.36	0.10	0.09	0.03	0.04	0.11	0.16	0.07	0.09	0.01	0.03	0.96	1.09
7	0.31	0.28	0.06	0.09	0.31	0.35	0.10	0.10	0.02	0.04	0.10	0.16	0.06	0.09	0.01	0.02	0.96	1.13
8	0.29	0.22	0.03	0.08	0.27	0.29	0.08	0.07	0.02	0.03	0.11	0.13	0.06	0.06	0.01	0.01	0.88	0.89
9	0.35	0.20	0.06	0.05	0.37	0.30	0.09	0.07	0.03	0.03	0.14	0.15	0.09	0.09	0.01	0.02	1.14	0.90
10	0.55	0.20	0.28	0.05	0.59	0.29	0.19	0.07	0.07	0.03	0.23	0.15	0.13	0.09	0.02	0.01	2.06	0.88
11	0.21	0.28	0.07	0.09	0.25	0.31	0.07	0.08	0.02	0.03	0.09	0.15	0.06	0.08	0.00	0.02	0.79	1.02
12	0.31	0.21	0.04	0.08	0.27	0.27	0.09	0.07	0.02	0.03	0.09	0.12	0.06	0.06	0.00	0.01	0.89	0.86
13	0.35	0.29	0.06	0.12	0.34	0.26	0.12	0.01	0.03	0.05	0.14	0.19	0.08	0.10	0.01	0.02	1.13	1.03
14	0.30	0.13	0.05	0.02	0.30	0.15	0.09	0.05	0.02	0.01	0.11	0.07	0.07	0.04	0.02	0.00	0.96	0.47
15	0.29	0.24	0.03	0.06	0.24	0.31	0.08	0.09	0.02	0.03	0.10	0.14	0.06	0.07	0.01	0.02	0.83	0.93
16	0.32	0.23	0.05	0.06	0.29	0.30	0.10	0.07	0.02	0.03	0.10	0.14	0.06	0.08	0.01	0.02	0.95	0.91
17	0.23	0.24	0.10	0.06	0.31	0.27	0.08	0.06	0.03	0.03	0.13	0.13	0.06	0.06	0.01	0.02	0.96	0.87
18	0.37	0.22	0.06	0.09	0.37	0.31	0.12	0.07	0.03	0.04	0.15	0.13	0.09	0.07	0.01	0.01	1.21	0.94

19	0.40	0.25	0.05	0.06	0.41	0.33	0.13	0.08	0.04	0.02	0.15	0.15	0.09	0.08	0.01	0.01	1.28	0.99
20	0.34	0.26	0.04	0.11	0.30	0.29	0.13	0.08	0.02	0.03	0.12	0.13	0.07	0.06	0.01	0.02	1.02	0.99
21	0.32	0.21	0.05	0.06	0.35	0.29	0.08	0.07	0.03	0.03	0.13	0.12	0.07	0.07	0.01	0.01	1.03	0.86
22	0.21	0.20	0.03	0.04	0.19	0.25	0.04	0.05	0.03	0.02	0.09	0.12	0.04	0.06	0.01	0.01	0.64	0.76
23	0.27	0.13	0.04	0.07	0.24	0.27	0.08	0.07	0.02	0.03	0.09	0.13	0.05	0.06	0.01	0.01	0.81	0.78
24	0.37	0.26	0.05	0.06	0.35	0.30	0.12	0.07	0.04	0.03	0.13	0.13	0.08	0.06	0.01	0.02	1.15	0.93
25	0.32	0.28	0.05	0.08	0.28	0.33	0.11	0.07	0.02	0.03	0.11	0.16	0.08	0.08	0.02	0.02	0.99	1.04
26	0.38	0.20	0.06	0.04	0.38	0.30	0.11	0.08	0.03	0.03	0.14	0.14	0.09	0.06	0.01	0.02	1.21	0.87
27	0.30	0.20	0.05	0.08	0.31	0.30	0.09	0.07	0.02	0.03	0.11	0.15	0.07	0.08	0.01	0.02	0.96	0.93
28	0.41	0.24	0.05	0.10	0.36	0.23	0.12	0.01	0.03	0.03	0.13	0.14	0.08	0.08	0.01	0.03	1.20	0.86
29	0.31	0.20	0.06	0.07	0.30	0.29	0.10	0.08	0.02	0.03	0.12	0.14	0.07	0.07	0.01	0.02	0.98	0.90
30	0.24	0.27	0.10	0.07	0.29	0.36	0.10	0.09	0.03	0.02	0.12	0.17	0.06	0.08	0.01	0.04	0.95	1.09
31	0.30	0.29	0.07	0.07	0.32	0.34	0.10	0.10	0.04	0.03	0.13	0.16	0.07	0.08	0.01	0.02	1.03	1.08

Table S4. Colour coordinates acquired in the 31 runs for UAE and MAE. Terms used include: L*: lightness; a*: green-red colour component; b*: blue-yellow colour component and C: Chroma.

Run	Colour coordinates							
	*L**		*a**		*b**		Chroma	
	UAE	MAE	UAE	MAE	UAE	MAE	UAE	MAE
1	43.82	36.12	83.84	76.12	-22.35	-0.99	86.77	76.12
2	45.36	49.71	84.50	74.89	-23.90	26.47	87.82	79.43
3	47.39	46.89	85.33	78.96	-26.95	18.42	89.49	81.08
4	48.17	37.51	83.07	77.84	-24.19	-3.40	86.52	77.92
5	45.50	38.62	84.51	76.27	-22.47	4.67	87.44	76.41
6	47.70	50.57	85.37	74.00	-24.99	24.80	88.95	78.05
7	43.99	37.31	83.30	74.05	-21.97	11.04	86.15	74.87
8	46.60	38.17	86.48	76.71	-25.64	3.88	90.20	76.80
9	45.84	45.24	85.24	73.07	-23.82	25.69	88.51	77.46
10	45.60	36.20	84.99	75.34	-23.10	3.32	88.08	75.41
11	45.93	38.43	85.18	73.83	-24.28	8.39	88.57	75.01
12	48.44	40.32	86.35	75.52	-26.97	13.48	90.46	76.72
13	48.81	37.01	86.25	77.75	-27.53	0.38	90.54	77.75
14	55.56	34.01	76.34	72.32	-23.63	0.36	79.91	72.33
15	44.73	36.78	83.56	77.28	-22.94	-1.61	86.65	77.30
16	48.48	35.41	86.56	73.50	-27.00	5.64	90.67	73.72
17	44.76	35.11	84.74	73.47	-22.46	6.12	87.66	73.73
18	43.87	35.24	83.12	74.33	-21.03	1.23	85.74	74.34
19	43.14	41.82	83.22	76.36	-21.64	16.74	85.99	78.18
20	44.36	35.54	84.80	74.08	-21.43	5.65	87.47	74.30
21	45.24	34.86	84.83	74.35	-22.22	0.36	87.69	74.36
22	43.76	34.09	83.32	72.65	-20.83	1.82	85.89	72.67
23	48.05	36.70	85.75	75.18	-24.05	3.41	89.06	75.26
24	45.35	35.46	84.89	73.99	-21.61	4.10	87.60	74.10
25	47.51	36.19	85.71	72.86	-22.68	8.00	88.65	73.29
26	44.18	37.42	83.35	73.53	-20.70	8.36	85.88	74.00
27	44.96	36.78	84.71	77.95	-21.22	-5.64	87.32	78.16
28	44.50	34.71	84.58	73.31	-20.55	5.84	87.04	73.54
29	46.12	34.93	84.76	59.35	-23.37	26.34	87.93	64.99
30	43.67	35.31	83.03	75.06	-19.85	-0.79	85.37	75.07
31	44.40	35.39	83.24	74.07	-19.78	5.28	85.56	74.26

Figure S1. Total betacyanin content 3d response surface plots of UAE and MAE for the parametric responses. For representation purposes, the 2 variable which are not confronted, were positioned at the centre of their experimental domain.

Betalains response surface graphs

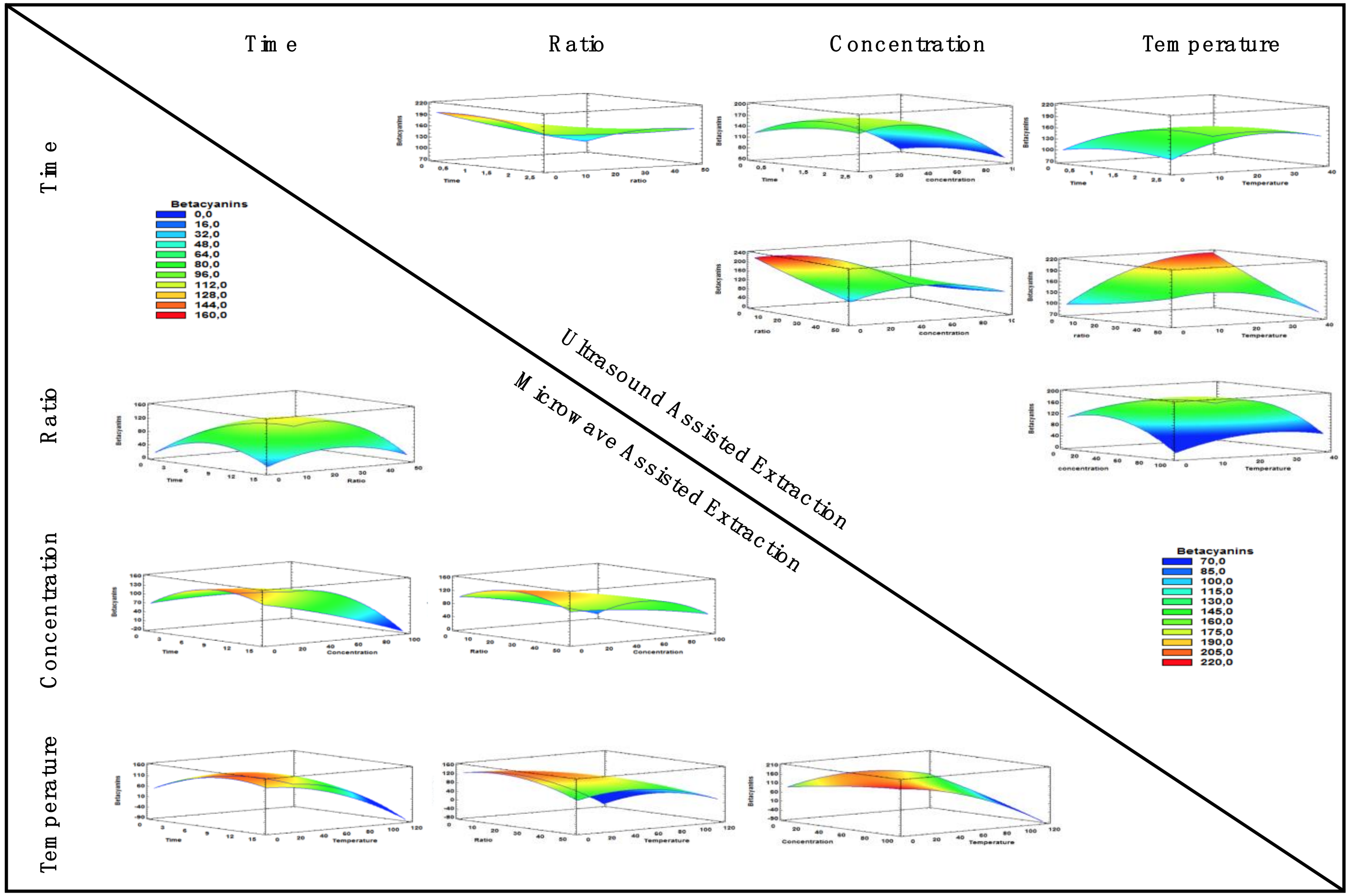

Figure S2. Total phenolic acid content 3d response surface plots of UAE and MAE for the parametric responses. For representation purposes, the 2 variable which are not confronted, were positioned at the centre of their experimental domain.

Phenolic compounds response surface graphs

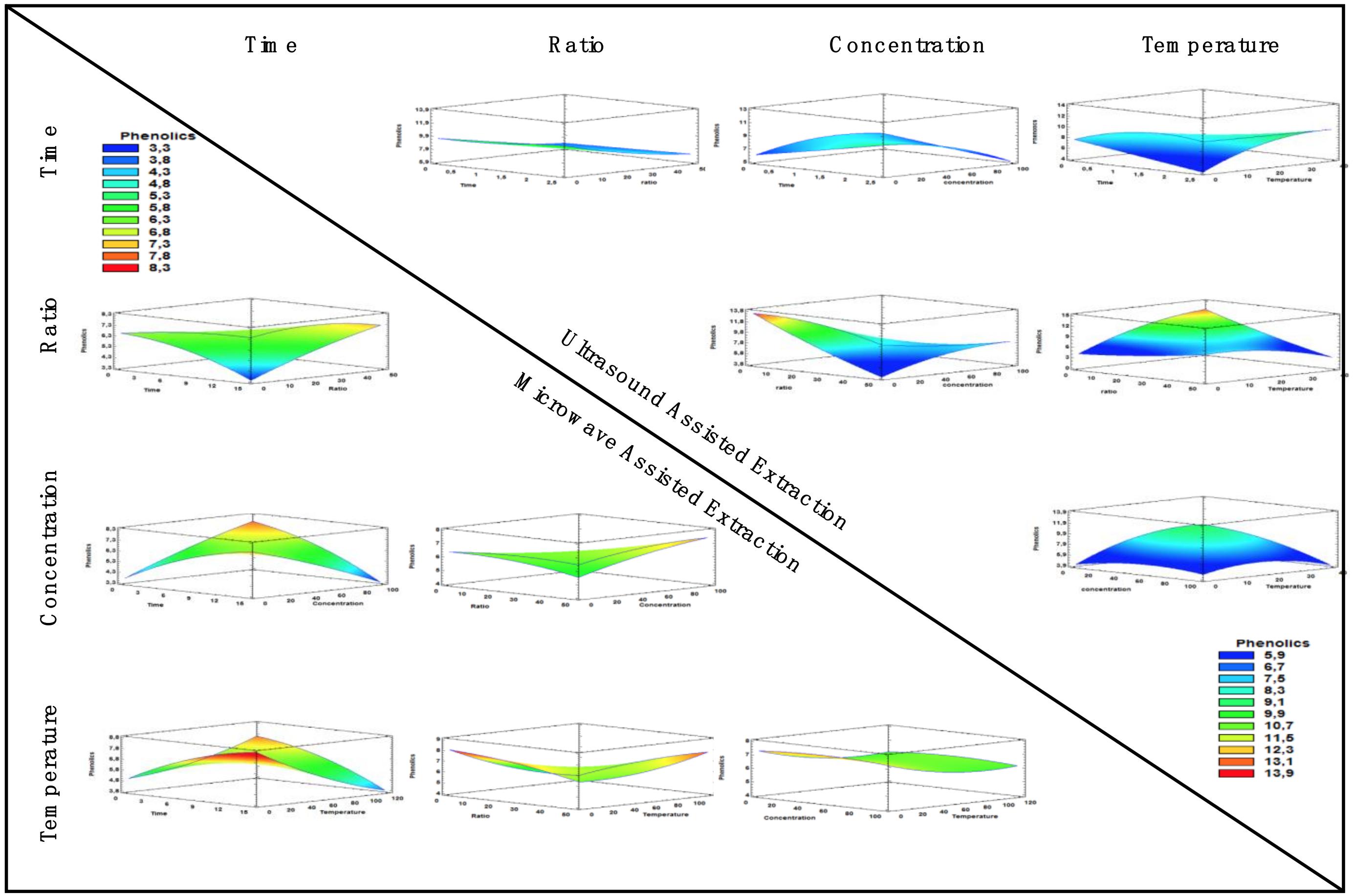

Figure S3. Total flavonoid content 3d response surface plots of UAE and MAE for the parametric responses. For representation purposes, the 2 variable which are not confronted, were positioned at the centre of their experimental domain.

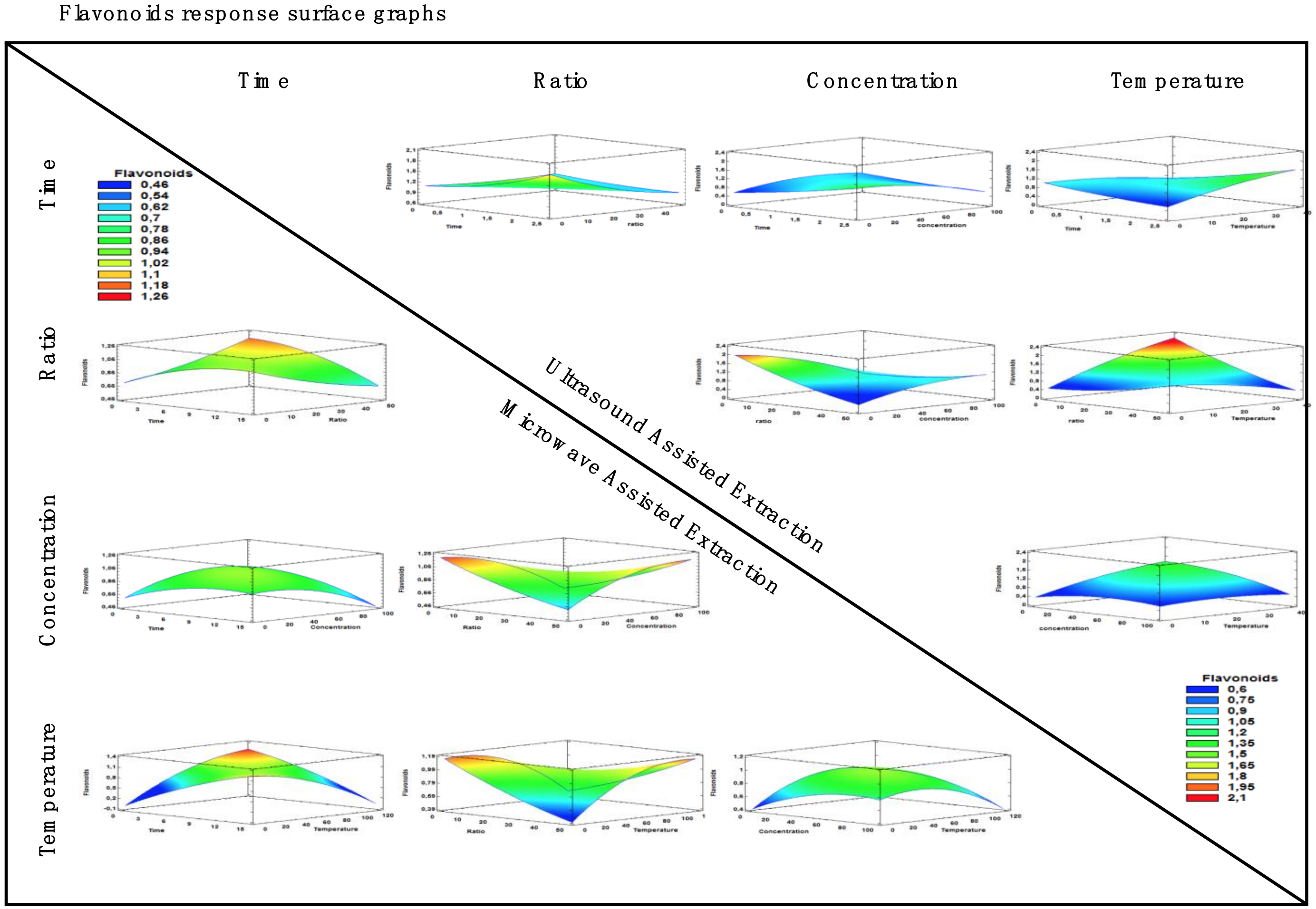

Figure S4. Antioxidant activity 3d response surface plots of UAE and MAE for the parametric responses. For representation purposes, the 2 variable which are not confronted, were positioned at the centre of their experimental domain.

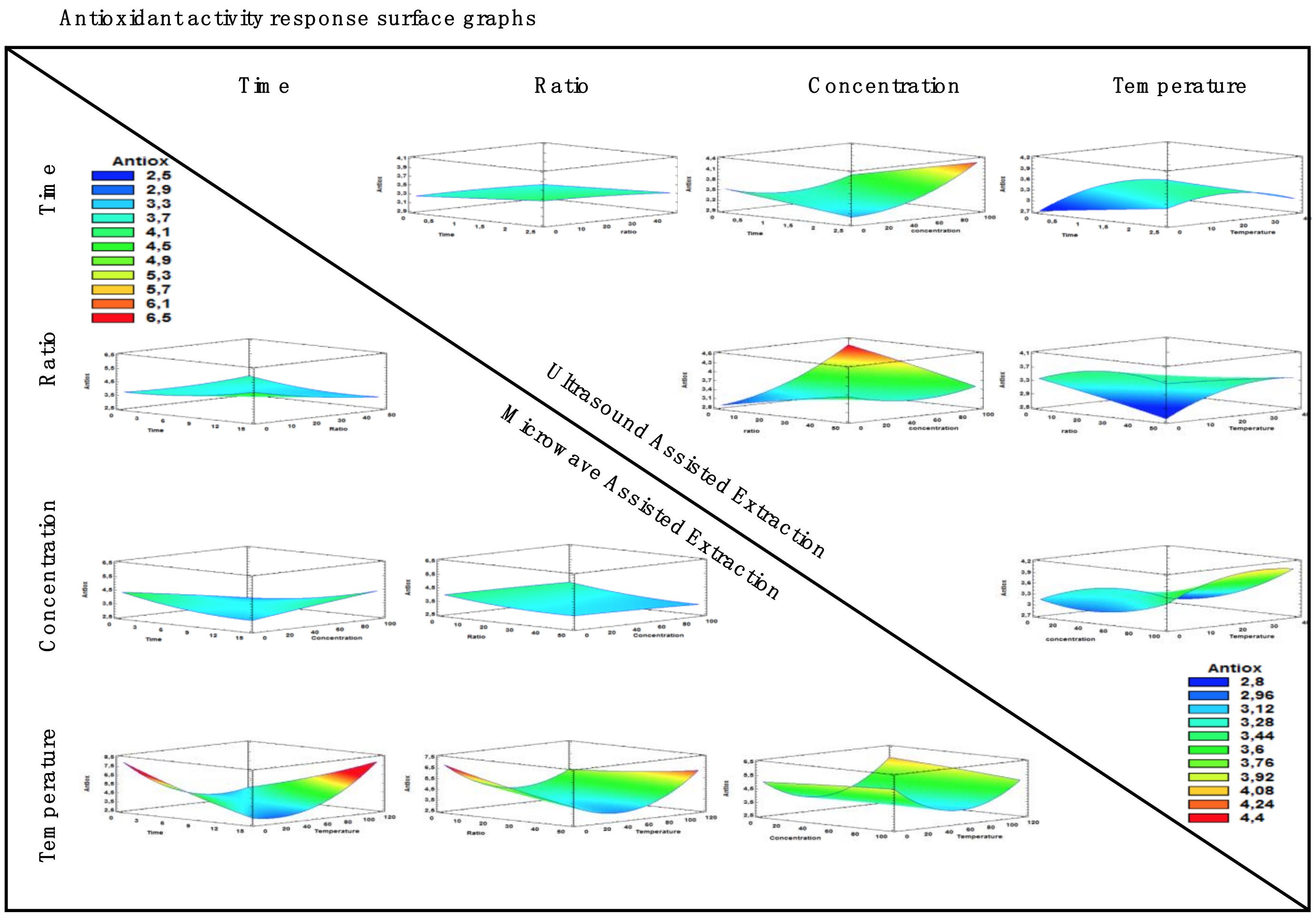

Supplement: Supplementary file 1 [file molecules-24-03618-s001.pdf]
